# Clinical Presentation, Renal Histopathological Findings, and Outcome in Patients with Monoclonal Gammopathy and Kidney Disease

**DOI:** 10.1155/2021/8859340

**Published:** 2021-05-12

**Authors:** Gaetano Alfano, Alice Delrio, Francesco Fontana, Giacomo Mori, Silvia Cazzato, Annachiara Ferrari, Rossella Perrone, Silvia Giovanella, Giulia Ligabue, Riccardo Magistroni, Gianni Cappelli

**Affiliations:** ^1^Surgical, Medical and Dental Department of Morphological Sciences, Section of Nephrology, University of Modena and Reggio Emilia, Modena, Italy; ^2^Nephrology Dialysis and Transplant Unit, University Hospital of Modena, Modena, Italy; ^3^General Medicine, Azienda Unità Sanitaria Locale, Modena, Italy

## Abstract

Monoclonal gammopathies are associated with acute and chronic kidney injury. Nephrotoxicity of the secreted monoclonal (M)-protein is related to its biological properties and blood concentration. Little is known about epidemiology, clinical manifestations, and outcome of monoclonal gammopathies in patients with kidney disease. We retrospectively collected data about demographics, clinical manifestations, and renal histological lesions of all patients (*n* = 1334) who underwent kidney biopsy between January 2000 and March 2017. Monoclonal gammopathy was detected in 174 (13%) patients with a mean age of 66.4 ± 13.1 years. The spectrum of monoclonal gammopathies comprised monoclonal gammopathy of undetermined significate (MGUS) (52.8%), multiple myeloma (MM) (25.2%), primary amyloidosis (AL) (9.1%), smoldering MM (SMM) (4%), non-Hodgkin lymphoma (NHL) (6.8%), and Hodgkin lymphoma (HL) (1.7%). Monoclonal gammopathy of renal significance (MGRS) accounted for 6.5% in patients with MGUS and 14.2% in patients with SMM. Evaluation of kidney biopsy revealed that M-protein was directly involved in causing kidney injury in MM (93.1%). MM was the only gammopathy significantly associated with an increased risk of kidney injury (odds ratio [OR] = 47.5, CI 95%, 13.7–164.9; *P* ≤ 0.001). While there were no significant differences in the progression toward end-stage renal disease or dialysis (*P* = 0.776), monoclonal gammopathies were associated with a different risk of death (*P* = 0.047) at the end of the follow-up. In conclusion, monoclonal gammopathy was a frequent finding (13%) in patients who underwent kidney biopsy. M-protein was secreted by both premalignant (56.8%) and malignant (43.2%) lymphoproliferative clones. Kidney biopsy had a key role in identifying MGRS in patients with MGUS (6.5%) and SMM (14.2%). Among monoclonal gammopathies, only MM was significantly associated with biopsy-proven kidney injury. The rate of end-stage renal disease or dialysis was similar among monoclonal gammopathies, whereas NHL, MM, and SMM showed a higher rate of deaths.

## 1. Introduction

Monoclonal gammopathy is a clinical condition characterized by the presence of an abnormal protein—known as monoclonal (M)-protein or paraprotein—in the blood [1]. M-protein is an intact antibody, or any chain fragment produced and secreted by a pathological lymphoproliferative clone. The ability of M-protein to disrupt cellular homeostasis is unpredictable. It is principally related to its physicochemical properties and blood concentration [2].

Historically, renal toxicity of the M-protein has been associated with the malignancy of the underlying lymphoproliferative disorder. Multiple myeloma (MM), one of the most common hematologic malignancies [3], has been widely associated with kidney disease [4, 5]. Renal injury in this disease relies principally on the overproduction of M-protein. This, freely filtered by the glomerulus, overwhelms tubular reabsorption capacity leading to intraluminal precipitation with tubular obstruction and activation of inflammatory pathways [6].

Other malignant lymphoproliferative diseases, such as Waldenström macroglobulinemia (WM) [7] and lymphomas [8, 9], have been frequently associated with renal injury and can present with a wide range of glomerular lesions including M-immunoglobulin deposition disease, proliferative glomerulonephritis with M-immunoglobulin deposit, and cryoglobulinemic vasculitis. Even clones, which have a low propensity to progress toward malignancy and secrete a low amount of M-protein, can be involved in tissue damage, including neuropathy and autoimmune diseases as well as kidney diseases [10]. In this setting, tissue deposition of M-protein (direct mechanism) or activation of the complement system without tissue deposition M-protein (indirect mechanism) may lead to kidney injury [11]. Recently, the nephrological community has coined a new term: monoclonal gammopathy of renal significance (MGRS). The definition of MGRS includes all small B-cell or plasma cell clones that, per se, do not meet strict hematological criteria for cytoreductive therapy but are implied in kidney injury through the production of M-protein [12]. The most glaring example is monoclonal gammopathy of undetermined significate (MGUS), which may be associated with renal lesions despite the low measureable levels of secreted paraprotein and the rare progression to MM [13]. The growing interest in this new pathological entity has spurred nephrologists and hematologists to reevaluate the pathogenicity of these small and apparently indolent clones. The enthusiasm to understand the causative role of M-protein in patients with renal impairment was tempered by the rarity of the phenomena. Considering these limits and the fragmentation of the data published in the literature, we explored the association between monoclonal gammopathies and kidney disease with the aim to define prevalence and clinical manifestations as well as outcomes of these disorders in a cohort of patients who underwent kidney biopsy for renal impairment.

## 2. Material and Methods

We conducted a retrospective study at the Nephrology Unit of the University Hospital of Modena. The medical charts of all patients with biopsy-proven kidney disease were evaluated from January 2000 to March 2017. Among all patients who underwent kidney biopsy, we enrolled only those with a diagnosis of serum M-protein that was detected by protein electrophoresis (SPEP) and subsequently characterized by serum or urine immunofixation. Hence, patients with an unconfirmed diagnosis of monoclonal gammopathies were excluded from the study. The study protocol was approved by the Provincial Ethics Committee of the University Hospital of Modena (CE/1476).

### 2.1. Renal Biopsy

Biopsy specimens were examined using light microscopy (LM) and immunofluorescence (IF). Kidney tissue sections have been evaluated by hematoxylin and eosin, periodic acid-Schiff (PAS), periodic acid-methenamine silver (Jones), and Masson's trichrome stains. For immunofluorescence, cryostat sections were stained with fluorescein isothiocyanate-conjugated rabbit anti-human IgG, IgM, IgA, C3, C1q, kappa (*k*), and lambda (*λ*) light chain. Staining with Congo red was used to confirm amyloid deposits. Primary amyloidosis or light chain amyloidosis (AL) was identified by light chain restriction using IF or immunohistochemistry performed in another center. Electron microscopy examination was performed only on pathologists' indication. Monoclonal gammopathy was considered directly involved in the pathogenesis of renal lesions if histological examination revealed immunoglobulin-associated kidney lesions with heavy or light chain restriction on IF (restriction for the same immunoglobulin isotype detected in the blood). Cast nephropathy, M-immunoglobulin deposition disease, membranoproliferative glomerulonephritis with M-immunoglobulin deposit, and primary amyloidosis or light chain amyloidosis (AL) were part of the group of M-protein-associated kidney diseases [11].

### 2.2. Data Collection and Definition

Demographics and laboratory data were collected at the time of kidney biopsy. Data about complete blood count (leukocytes, erythrocytes, hemoglobin, and platelets), serum calcium, serum creatinine (sCr), estimated glomerular filtration (eGFR) (calculated using CKD-EPI equation) [14], proteinuria, serum and urine M-protein, serum M-protein concentration, and serum-free light chain (FLC) were extracted from medical records.

Proteinuria was principally estimated through the urine protein-to-creatinine ratio.

Nephrotic syndrome was defined as urine protein-to-creatinine ratio greater than 3 mg/mg and serum albumin less than 3.5 gr/dl. We considered AL amyloidosis and light chain deposition disease associated with MM in the presence of lytic bone lesions and other signs of over MM or plasma cells count greater than 30% in the bone marrow [15].

Acute kidney injury (AKI) referred to an abrupt decrease in kidney function. The definition is based on the following criteria: increase in sCr by ≥0.3 mg/dL within 48 hours, or increase in sCr level to ≥1.5 times from baseline [16]. The urinary criterium was not used for the diagnosis of AKI. Baseline sCr corresponded to the last sCr before kidney biopsy. When it was not available, we considered sCr measured at hospital admission.

Severe impairment of renal function referred to acute worsening of renal function with a serum sCr level ≥3 mg/dL.

Bone marrow biopsy was performed according to the hematologist's indications after identification and characterization of the serum M-protein, radiologic workup, and flow cytometry.

### 2.3. Statistical Analysis

Continuous variables with normal distribution were presented as mean and standard deviation (SD). The difference between the means of two groups was performed with a two-sample *t*-test or Mann–Whitney test.

One-way ANOVA and Kruskal–Wallis test (or one-way ANOVA on ranks) were used to perform multiple comparisons between groups. Tukey's test and χ2 test or Fisher's exact test were used to determine the statistical difference between the mean of all possible pair and categorical data, respectively. Logistic regression analysis was done to compute odds ratios (OR), 95% confidence intervals (95% CI), and *P* values of lymphoproliferative disorders in predicting direct kidney injury. Logistic regression was also used to evaluate the association between histological findings in MM patients and severe renal impairment. *P* value <0.05 was considered to be statistically significant. All analyses were performed using SPSS version 23 (SPSS, Inc., Chicago, IL).

## 3. Results

### 3.1. Patient's Characteristics

We reviewed the charts of 1334 patients who underwent native kidney biopsy for renal dysfunction. Monoclonal gammopathy was found in 174 patients (13%) with a mean age of 66.4 ± 13.1 years. Most of them were of Caucasian origin (96%), and males were predominant over females (67.2 vs. 32.8%) (Table 1). Compared to the entire study population, monoclonal gammopathy was more frequent in patients aged 50–79 years (Table 2).

**Figure 1:**
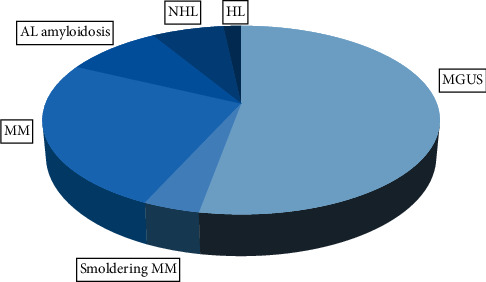
Distribution of monoclonal gammopathy in our cohort of patients.

The hematologic disorders presenting with M-protein in peripheral blood were MGUS (52.8%), MM (25.2%), AL amyloidosis (9.1%), non-Hodgkin lymphoma (NHL) (6.8%), smoldering MM (SMM) (4%), and Hodgkin lymphoma (HL) (1.7%) (Table 1 and Figure 1). The detection of M-protein was associated with 56.8% of premalignant lymphoproliferative diseases and 43.2% of malignant diseases. There were no differences (*P* = 0.16) between the age of patients with benign (5.3 ± 13.7 years) and malignant lymphoproliferative diseases (67.5 ± 11.9 years).

### 3.2. MGUS

MGUS was the most common monoclonal gammopathy. Its prevalence was estimated to be 52.8% among patients with serum M-protein.

The mean age of patients was 64.9 ± 13.9 years with predominance of males of 62% (Table 3).

In 16 (17.3%) patients, the histological evaluation revealed glomerular lesions compatible with a membranoproliferative glomerulonephritis pattern. It was secondary to viral hepatitis in 4 subjects. Further kidney diseases included membranous glomerulopathy (16.3%), end-stage kidney disease (ESRD) (16.3%), ANCA-associated vasculitis (8.6%), postinfectious glomerulonephritis (7.6%), focal segmental glomerular sclerosis (5.4), and interstitial nephritis (4.3%). Surprisingly, IgA glomerulonephritis was underrepresented in this group (1%).

In four patients (4.3%), MGUS was directly involved in the development of kidney injury through the deposition of light chains. In two cases, IF analysis showed intact immunoglobulin restriction in a setting of membranoproliferative pattern of glomerular injury. Overall, parenchymal lesions compatible with MGRS criteria accounted for 6.5% of all cases of MGUS.

At the end of the observational period of 5.4 years, 37% of patients died (Table 4) and 32% were on replacement renal therapy (Table 5).

### 3.3. Multiple Myeloma

MM was the second most common gammopathy in our study population. The disorder was detected in 44 subjects with a mean age of 66.9 ± 12.9 years. MM was more frequent in males than in females (72.7 vs. 27.3%). According to the criteria CRAB (hypercalcemia, renal disease, anemia, and bone disease), laboratory tests at presentation revealed serum calcium of 9 ± 1.1 mg/dl, hemoglobin of 10.2 ± 1.6 gr/dl, myeloma bone lesions in 75% of patients, and average sCr of 4.3 ± 2.9 mg/dl, corresponding to an eGFR of 28.4.7 ± 28.9 ml/min (Table 3). Plasma cells infiltrate in bone marrow biopsy accounted for 50% of the cells. The majority of the patients (81.8%) was admitted with severe impairment of renal function and 27.2% needed urgent kidney replacement therapy. In seven patients (15.9%), clinical manifestation of kidney disease was nephrotic syndrome with average urine protein-to-creatinine ratio ranging from 4.2 to 18.5 mg/mg associated with a wide variability of renal function (sCr ranged from 1.01 to 6 mg/dl).

Light chain MM accounted for 34.1% of the cases and, as expected, k light chain MM was more prevalent compared to *λ* light chain MM (60% vs. 40%). M-protein isotypes were IgGk (22.7%), IgG*λ* (15.9%), IgAk (6.8%), IgA*λ* (13.6%), and IgMk (6.8%). Bence Jones protein was detected in 93.1% of the tested patients.

Histological evaluation of kidney biopsy specimens showed cast nephropathy (68.1%), AL amyloidosis (15.9%), light chain deposition disease (6.8%), and interstitial nephritis (9.1%). Cast nephropathy was the only histological lesion associated with severe renal impairment (OR = 26.2, 95% CI, 2.8–245.5; *P* = 0.004) (Supplemental Table 1). Lastly, all patients with histological diagnosis, different from cast nephropathy, had bone osteolytic lesions compatible with myeloma bone disease. The case fatality rate was high (52.3%) and more than one-third of the patients were on ESRD (38.2%) at the end of the follow-up period (Tables 4 and 5).

### 3.4. Smoldering Multiple Myeloma

Seven (4%) patients had a diagnosis of SMM. The disorder manifested at a mean age of 71.8 ± 11.9 years and showed a slightly higher prevalence in men than women (72.7% vs. 27.3%).

According to the definition of SMM, bone lytic lesions were absent in all patients. Hemoglobin and serum calcium were in the normal range, 12.3 ± 2.3 gr/dl and 8.6 ± 0.8 mg/dl, respectively (Table 3). At presentation, mean sCr was 2.7 ± 2.9 mg/dl (eGFR of 43.5 ± 30.3 ml/min) with proteinuria of 1.4 ± 1.1 mg/mg.

Immunofixation of the serum M-protein detected the following isotypes: IgG*λ* (28.2%), IgGk (28.2%), IgMk (14.1%), IgA*λ* (14.1%), and k light chain (14.1%). Bence Jones protein was found in 71.4% of the patients. Bone marrow biopsy revealed a mean plasma cell count of 18%.

Evaluation of renal biopsies showed different patterns of glomerular diseases including membranoproliferative glomerulonephritis (28.5%), interstitial nephritis (14.2%), light chain deposition disease (14.2%), acute tubular necrosis (ATN) (14.2%), ANCA-negative vasculitis (14.2%), and membranous glomerulonephritis (14.2%). Light chain restriction was diagnosed only in one patient (14.2%) affected by membranoproliferative glomerulonephritis.

### 3.5. Hodgkin Lymphoma

Three patients (1.12%) had a diagnosis of HL at an average age of 69.04 ± 5.3 years. All patients had a normal renal function manifesting with a mean sCr of 0.93 ± 0.07 mg/dl corresponding to an eGFR of 62.7.3 ± 7.4 ml/min. Mean urine protein-to-creatinine ratio was 0.3 ± 0.2 mg/mg (Table 3). Mild proteinuria was present in only one patient (urine protein-to-creatinine ratio of 0.5 mg/mg). Bence Jones protein was present in only one patient. Cryoglobulinemic glomerulonephritis was found in two-thirds of the patients (66.6%) and hypertensive nephrosclerosis in one (33.3%).

### 3.6. AL Amyloidosis

Amyloidosis, defined as primary amyloidosis or AL amyloidosis, was diagnosed in 16 patients (9.1%). Amyloidosis was secondary to MGUS (75%) and SMM (25%). The mean age of the affected subjects was 66.34 ± 11.38 years and females were slightly more prevalent than males (53 vs. 46%).

sCr ranged from 0.5 to 4.5 mg/dl with a mean level of 1.4 gr/dl, corresponding to 56.5 ml/min of eGFR. Nephrotic syndrome was the most common presentation (75%). Overall, patients presented with high proteinuria (8.33 ± 3.2 mg/mg) associated with hypoalbuminemia (2.74 ± 0.84 gr/dl) (Table 3).

The diagnosis of amyloidosis was performed by the detection of deposit of amorphous material in the mesangium and capillary loops of glomeruli. Congo red stain confirmed the diagnosis of amyloidosis and immunohistochemical analysis identified the corresponding serum light chain.

### 3.7. Non-Hodgkin's Lymphoma

Twelve patients (6.8%) with monoclonal gammopathy had a diagnosis of NHL, whose term includes several heterogeneous lymphoproliferative disorders. According to the WHO classification [17], lymphoplasmacytic lymphoma accounted for 41.6%, Waldenstrom's macroglobulinemia for 30.7%, marginal zone lymphoma for 15.2%, diffuse large B-cell lymphoma for 7.6%, and anaplastic large cell lymphoma for 7.6%. Male gender was fully associated (100%) with NHL in our cohort of patients. The average age of subjects was 72.6 ± 9.6 years. Renal function was extremely variable at presentation, with sCr ranging between 0.85 and 6.35 mg/dl; mean sCr was 2.4 ± 1.6 mg/dl corresponding at an eGFR of 30.4 ± 22.7 ml/min. Five out of 12 patients had nephrotic syndrome at hospital admission. Daily proteinuria ranged from 0.7 to 8.2 mg/mg, and mean proteinuria was 4.36 ± 3.36 mg/mg/24 hours.

IgMk(66.6%) was the most common monoclonal protein, whereas IgAλ, IgGk, and IgM*λ* accounted for 8.33%. A patient with marginal zone lymphoma had a circulating biclonal M-protein.

NHL subjects had a circulating M-protein of 0.6 ± 0.4 gr/dl. Urine monoclonal component was found in 68.7% of patients.

Histological evaluation of biopsy specimen revealed amyloidosis (25%), glomerular injury with membranoproliferative pattern (16.6%), LCDD (25%), ANCA-associated vasculitis (8.3%), cast nephropathy (8.3%), and hypertensive nephrosclerosis (8.3%). In one case (8.3%) Ig deposits were restricted for the same serum M-protein in a context of a membranoproliferative pattern of glomerular injury.

### 3.8. Comparison between Groups

Kruskal–Wallis test showed that mean serum sCr levels (*P* ≤ 0.0001), eGFR (*P*=0.004), proteinuria (*P* ≤ 0.042), serum calcium (*P* ≤ 0.0001), serum albumin (*P* ≤ 0.0001), white blood count (*P* ≤ 0.006), and hemoglobin (*P* ≤ 0.002) were statistically different between the groups (Table 3).

Lymphoproliferative diseases were variably associated with renal lesions due to M-protein. Documented M-protein-associated kidney injury accounted for 58.3%, 6.5%, 91.3%, 14.1%, and 100% in patients with NHL, MGUS, MM, SMM, and AL amyloidosis, respectively (Table 6). Excluding AL amyloidosis, regression analysis showed that MM was significantly associated with a 47.5-fold increased risk of renal lesions (95% CI, 13.7–164.9; P ≤ 0.001) .

The rate of ESRD or dialysis (*P* = 0.74) and death (*P* = 0.11) was not statistically significant in patients with monoclonal gammopathies at the end of follow-up. There was a trend toward high mortality in patients with SMM (71.4%), NHL (66.7%), and MM (52.3%).

**Figure 2:**
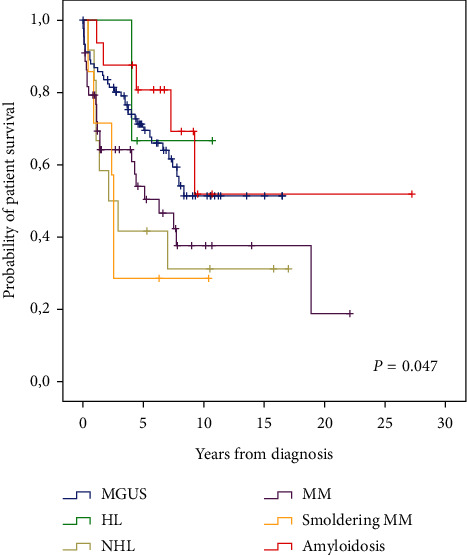
Overall survival according to the diagnosis of monoclonal gammopathy.

**Figure 3:**
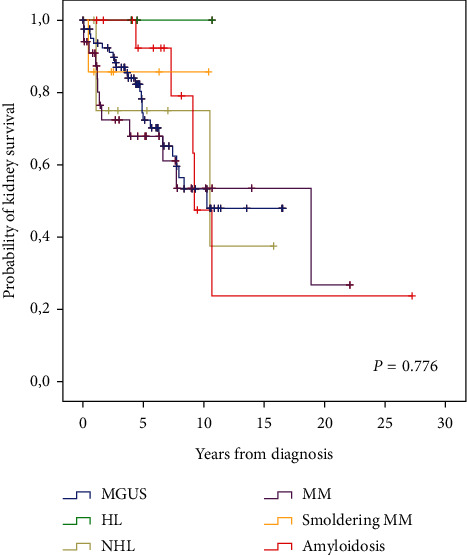
Death-censored kidney survival (CKD 5 or dialysis) according to the diagnosis of monoclonal gammopathy.

There were no statistically significant differences in crude case-rate fatality (*P* = 0.113) and incidence of ESRD or dialysis (*P* = 0.751) among groups with monoclonal gammopathies. Kaplan–Meier survival analysis revealed a statistically significant difference in the survival of patients with monoclonal gammopathy (*P* = 0.047) (Figure 2) and confirmed that there were no differences in the incidence of ESRD or dialysis (Figure 3).

## 4. Discussion

The recent literature has placed great emphasis on the pathogenic role of monoclonal gammopathy as a potential cause of kidney disease. Our study showed that monoclonal gammopathy was a frequent diagnosis (13%) in patients with renal impairment who underwent kidney biopsy. Monoclonal gammopathy occurred predominantly in subjects aged more than 50 years with a peak over 70 years. In our cohort of patients, we diagnosed MGUS, SMM, NHL, LH, MM, and AL amyloidosis. MGUS was the most common disorder. It accounted for more than half (52.8%) of all monoclonal gammopathies. Nevertheless, the prevalence rate of malignant lymphoproliferative disorders was surprisingly high in our cohort of patients since MM, HL, and NHL accounted for 42.8% of all gammopathies.

Besides the malignancy of these disorders, the nephrotoxicity of M-protein should be considered when evaluating monoclonal gammopathy. M-protein, may be extremely harmful to renal parenchyma, even though it is secreted by an indolent clone. Etiological mechanisms of M-protein nephrotoxicity are strictly dependent on the idiosyncratic properties of the secreted paraprotein. Deposition of M-protein [18] and activation of complement [19] are the leading pathological processes underlying the onset of monoclonal gammopathy-associated renal lesions [2].

According to the recent definition of MGRS [12], renal lesions due to the interplay with circulating M-protein were detected in 6.5% and 14.1% of MGUS and SMM patients, respectively. Histological lesions compatible with MGRS included light chain deposition diseases and proliferative glomerulonephritis with M-immunoglobulin deposits. While light chain deposition disease is known to be associated with the deposition of circulation of M-protein [20], little is known about the role of M-protein in promoting membranoproliferative glomerulonephritis [21]. This latter histologic pattern has been frequently encountered in patients with MGUS, but it is not uncommon in chronic lymphocytic leukemia, lymphomas, and MM [21]. Deposition of secreted monotypic immunoglobulin protein along the capillary walls and the activation of the complement system (both classical and alternative pathway) are believed to be the main triggers for the development of the membranoproliferative pattern of glomerular damage [21].

It is worth noting that the histological detection of glomerular lesions with membranoproliferative patter is not sufficient to meet the diagnosis of MGRS. The identification of the restricted circulating immunoglobulin in renal parenchyma by immunofluorescence (or immunoperoxidase) and transmission electron microscopy is a practical and effective way to demonstrate direct M-protein nephrotoxicity [12]. Although the membranoproliferative pattern of glomerular injury was the predominant histopathological finding in MGUS and SMM patients, we found a few cases with Ig-restriction, corresponding to about one-tenth of all patients with these lesions. 

Among all lymphoproliferative disorders, MM was significantly associated with direct kidney injury (*P* ≤ 0.0001). The etiological mechanism underlying kidney dysfunction was the production of a great amount of M-protein directly involved in the pathogenesis of myeloma-associated kidney disease. The majority of the patients with MM (81.8%) were admitted with AKI requiring renal replacement treatment in about a third of cases. In line with previous native renal biopsy studies [22, 23], cast nephropathy was the most prevalent histopathological finding (68.1%), and as expected, it was also significantly associated with severe renal impairment. Interestingly, tubulointerstitial nephritis, a rare renal manifestation of MM, was found to be 9% of all cases [24].

Lymphoma can be associated with kidney involvement presenting with a wide spectrum of manifestations. Lymphocytic infiltration of the parenchyma is the most prevalent finding in the largest case series of autopsies [25]. Further kidney manifestations rely on several distinct malignancy-related mechanisms and include minimal change disease, amyloidosis, membranoproliferative glomerulonephritis, immunotactoid glomerulopathy, and M-protein deposition disease. In the setting of HL, presentation of kidney involvement in our series was mild proteinuria with normal renal function, but the limited number of cases does not allow us to generalize thesedata. On the other hand, renal function was extremely variable in NHL patients, ranging from normal renal function to acute kidney failure. Glomerulonephritis with membranoproliferative-like patterns and M-immunoglobulin deposition disease were the most common histological findings in this group of patients. Similar to the literature, glomerular involvement with membranoproliferative glomerulonephritis [26, 27] and M-protein deposition disease [9, 28, 29] was common in patients with NHL [9, 30].

AL amyloidosis represented only a small percentage (8.9%) of all monoclonal gammopathies. AL amyloidosis was characterized by the deposition of light chain deposition in the renal parenchyma of all renal biopsy specimens [31]. Our results confirmed the high prevalence of *λ* light chain isotype. AL amyloidosis manifested with a nephrotic syndrome characterized by a significantly higher level of proteinuria than other gammopathies. Renal function was not severely impaired and showed only a slight increase in sCr.

At the end of the follow-up, the evolution of renal function was extremely heterogeneous. The rate of ESRD or dialysis ranged from 0% (HL) to 38.2% (MGUS) without statistically significant differences among the groups of patients. In particular, renal outcome of MM patients was less dramatic than the initial stage of the disease. Recovery of renal function occurred in many of them, and the prevalence of ESRD or dialysis did not increase after 4.4 ± 5 years of follow-up. Seven subjects with MGRS had a different renal prognosis at the end of the observation period; indeed in only two cases, CKD progressed to renal failure.

Survival of patients with malignant gammopathies was poorer than patients with a premalignant clone. However, multiple factors (underlying kidney disease, disease-specific therapies, and supportive care) may have influenced the outcomes of these patients. In particular, the prognosis of patients with malignant disorders is changed in the past 10–15 years with the administration of promising therapeutic strategies such as proteasome inhibitor bortezomib, monoclonal antibodies, and the immunomodulatory drugs such as thalidomide and lenalidomide [32, 33].

In clinical practice, the workflow process for the assessment of monoclonal gammopathy-associated renal lesions is based on the identification of the hematological disorder and underlying nephropathy. Once M-protein has been identified and characterized, exclusion of a malignant disorder should remain a high priority among nephrologists, as the outcome of the patient is associated with a poor prognosis if left untreated. Diagnostic tests such as flow cytometry, bone marrow biopsy, and radiological examinations should have a low threshold if there is a high suspicion for lymphoproliferative disease. Evaluation of renal function trajectory and urinary abnormalities is essential to assess renal function. Kidney biopsy has a crucial role in the diagnosis of the underlying hematological disorder and renal injuries driven by M-protein. Lastly, kidney biopsy carries important therapeutic and prognostic implications in subjects with MGRS, as this condition is associated with a concerning poor renal outcome and with a high rate of recurrence after renal transplantation [34].

The main limitations of the study are the retrospective analysis, the different follow-up duration, and the small sample size of certain groups of patients with rare monoclonal gammopathies (especially HL and SMM) that do not allow us to generalize our results. The not routine use of electron microscopy has potentially led to the underestimation of some cases of MGRS and points out an unintentional bias frequently present in the current literature. The data collected over 17 years underlines the difficulty to categorize some kidney biopsies reporting a diagnosis of “membranoproliferative glomerulonephritis.” This term now refers to a histological pattern of glomerular lesions rather than a diagnosis of kidney disease. To avoid misclassification, we classified all the diagnosis of “membranoproliferative glomerulonephritis” with the term “glomerular injury with membranoproliferative pattern.”

## 5. Conclusion

Lymphoproliferative disorders secreting M-protein carry a different potential for kidney injury. MGUS is the most frequent monoclonal gammopathy (52,8%) among patients who undergo kidney biopsy. Although MGUS has a low propensity to progress toward malignant disease, it is related to the development of MGRS (6.5%). MM is significantly associated with renal impairment and commonly manifesting with severe impairment of renal function. Patients diagnosed with AL amyloidosis had a higher level of proteinuria compared to the other monoclonal gammopathies. Careful evaluation is mandatory to identify malignant monoclonal disorders and MGRS because both conditions require specific chemotherapy treatment and have a different prognosis compared to other monoclonal gammopathies.

## Figures and Tables

**Table 1:** Demographics and clinical characteristics of patients with monoclonal gammopathy.

Characteristic	All patients
Age, yr	
Mean (±SD)	66.4 ± 13.1
Male *n* (%)	112 (67.2%)
Follow-up, year	5.3 ± 4.5

Ethnic group, *n* (%)	
Caucasian	167 (96)
Hispanic	1 (0.6)
Asiatic	3 (1.7)
African	3 (1.7)

Monoclonal gammopathy	
MGUS, *n* (%)	92 (52.8)
MM, *n* (%)	44 (25.2)
AL amyloidosis, *n* (%)	16 (9.1)
NHL, *n* (%)	12 (6.8)
SMM *n* (%)	7 (4)
HL, *n* (%)	3 (1.7)

HL, Hodgkin lymphoma; NHL, non-Hodgkin lymphoma; MGUS, monoclonal gammopathy of indeterminate significance; MM, multiple myeloma; SD, standard deviation; SMM, smoldering multiple myeloma.

**Table 2:** Range of age in patients with monoclonal gammopathy.

Range of age	Study population-*n*.	Patients with MG-*n*. (%)	MG patients/study population-%
≤50	572	24 (13.7)	4.1
50–59	63	22 (12.6)	24.0
60–69	232	49 (28.1)	0.1
70–79	241	64 (26.5)	26.6
≥80	226	15 (6.6)	6.6
Total	1334	174 (100)	13

HL, Hodgkin lymphoma; NHL, non-Hodgkin lymphoma; MGUS, monoclonal gammopathy of indeterminate significance; MM, multiple myeloma; SMM, smoldering multiple myeloma.

**Table 3:** Clinical characteristics and lab examinations of patients with monoclonal gammopathy.

Laboratory tests	MGUS	SMM	MM	Amyloidosis	NHL	HL	*P* value
Age (yr)	64.9C13.9	71.8 ± 11.9	66.9 ± 12.9	66.3 ± 11.3	72.6 ± 9.6	69 ± 5.3	0.377
Male *n* (%)	57 (62)	4 (57.1)	32 (72.7)	8 (50)	10 (83.3)	1 (33.3)	0.277
Follow-up (yr)	5.4 ± 3.7	3.6 ± 3.5	4.4 ± 5	7.6 ± 5.8	5.4 ± 5.9	6.3 ± 3.7	0.223
White blood cells (±SD) (per mm^3^)	7.5 ± 3.4^∗^	6.3 ± 1.5^#^	6.2 ± 2^†^	7.1 ± 2.5^‡^	7.6 ± 3	14.1 ± 16^∗,#,†,‡^	**0.006**
Hemoglobin (±SD) (gr/dl)	11.3 ± 2.5	12.3 ± 2.7	10.2 ± 1.6^∗^	12.7 ± 2.0^∗^	11.3 ± 2	13.3 ± 0.9	**0.002**
Platelets (±SD) (per mm^3^)	219 ± 102.6^∗^	193.2 ± 47.8	209 ± 112.3^#^	306.6 ± 154.8^∗,#^	245 ± 39.2	188.7 ± 52.6	0.46
Albumin (±SD) (g/dl)	3.1 ± 0.8^∗^	3.6 ± 0.7	5.4 ± 3^∗,#,†^	2.8 ± 0.7^†^	3.4 ± 0.6^#^	3.7 ± 0.7	**<0.001**
sCr (±SD) (mg/dl)	2.68 ± 2.1^∗^	2.7 ± 2.9	4.3 ± 2.9^∗,#^	1.4 ± 1^#^	2.4 ± 1.6	0.93 ± 0.1	**<0.001**
eGFR (±SD) (ml/min)	35.2 ± 29.3	43.5 ± 30.3	28.4 ± 28.9^∗^	61.6 ± 31.1^∗^	39.9 ± 30.4	62.7 ± 7.4	**0.004**
Ca (±SD) (mg/dl)	8.5 ± 1^∗^	8.6 ± 0.8^#^	9 ± 1.1^∗,#,†^	9 ± 2.1	8 ± 1	9.6 ± 0.6^†^	**<0.001**
Urine protein-to-sCr ratio (±SD)	5.1 ± 6.5	1.4 ± 1.1	3.2 ± 4.6^∗^	13.5 ± 7.5^∗^	1.6 ± 2.9	0.3 ± 0.2	**0.042**
Serum M-protein (±SD) (gr/dl)	0.6 ± 0.5	0.8 ± 0.7	1.12 ± 1	0.7 ± 0.5	0.6 ± 0.4	NA	0.5
FLC *k* (±SD) (mg/dl)	302 ± 177.8	228.5 ± 161.5	411.5 ± 514.4	140 ± 101.5	319.3 ± 178.1	254 ± 173.1	0.08
FLC *λ* (±SD) (mg/dl)	172.5 ± 159.3	260.6 ± 196.2	187.2 ± 231.1	200.4 ± 208.7	148.8 ± 115.2	140.8 ± 117.9	0.868
Urine M-protein (%)	28.2^*∗*^	71.4	100^#,∗,†,‡^	68.7^#^	50^†^	33.3^‡^	**<0.001**

CKD, chronic kidney disease; eGFR, estimated glomerular filtration rate; FLC, free light chain; HL, Hodgkin lymphoma; NHL, non-Hodgkin lymphoma; MGUS, monoclonal gammopathy of indeterminate significance; MM, multiple myeloma; M-protein, monoclonal protein; sCr, serum creatinine; SD, standard deviation; SMM, smoldering multiple myeloma. The symbols ^∗,#,†^, and ‡ indicate statistical significance (*P* ≤ 0.05) among the single variables.

**Table 4:** Case fatality rate in patients with monoclonal gammopathy.

	MGUS (*n* = 92)	SMM (*n* = 7)	MM (*n* = 44)	Amyloidosis (=16)	NHL (*n* = 12)	HL (*n* = 3)	Total
Survival-*n.* (%)	58 (63)	2 (28.6)	21 (47.7)	11 (68.8)	4 (33.3)	2 (66.7)	98 (56.3)
No survival-*n.* (%)	34 (37)	5 (71.4)	23 (52.3)	5 (31.3)	8 (66.7)	1 (33.3)	76 (43.7)

**Table 5:** Rate of end-stage renal disease or dialysis in patients with monoclonal gammopathy.

	MGUS	SMM	MM	Amyloidosis	NHL	HL	Total^*∗*^
	(*n* = 92)	(*n* = 7)	(*n* = 44)	(=16)	(*n* = 12)	(*n* = 3)	
CKD stages 1–4-*n.* (%)	57 (67.9)	5 (83.3)	21 (61.8)	11 (68.8)	6 (66.7)	3 (100)	103 (67.8)
CKD stage 5/dialysis-*n.* (%)	27 (32.1)	1 (16.7)	13 (38.2)	5 (31.3)	3 (33.3)	0 (3)	49 (32.2)

^*∗*^Missing data = 13%. CKD, chronic kidney disease; HL, Hodgkin lymphoma; NHL, non-Hodgkin lymphoma; MGUS, monoclonal gammopathy of indeterminate significance; MM, multiple myeloma; SMM, smoldering multiple myeloma.

**Table 6:** Association between monoclonal gammopathies and direct kidney injury.

Monoclonal gammopathy	Odds ratio (95% CI)^∗^	*P* value
MM	47.5 (13.7–164.9)	**<0.001**
NHL	2.1 (0.6–7.2)	0.194
SMM	0.5 (0.1–3)	0.5
MGUS	0.01 (0.005–0.04)	<0.001
HL	—	0.153

HL, Hodgkin lymphoma; NHL, non-Hodgkin lymphoma; MGUS, monoclonal gammopathy of indeterminate significance; MM, multiple myeloma; SMM, smoldering multiple myeloma. AL amyloidosis was not tested because the pathogenesis of amyloidosis depends on a proven direct kidney injury due to the deposition of light chain within the renal parenchyma. *P* value for MGUS was statistically but not clinically significant. The protective effect of MGUS in developing kidney disease is not applicable in this context.

## Data Availability

The data will be shared on request to the corresponding author. Please contact Dr. Gaetano Alfano (e-mail: gaetano.alfano@unimore.it) to request them.

## Supplementary Materials

Supplementary Materials Supplemental Table 1: prediction of severe renal impairment (serum creatinine ≥ 3 mg/dl) according to renal histological findings found in kidney biopsy of MM patients.
