# Supplementary material for: Clinical Presentation, Renal Histopathological Findings, and Outcome in Patients with Monoclonal Gammopathy and Kidney Disease
Source: Int J Nephrol. 2021 May 12;2021:8859340. doi: 10.1155/2021/8859340 (PMC8137312; doi:10.1155/2021/8859340)
Supplement: Supplementary Materials — Supplemental Table 1: prediction of severe renal impairment (serum creatinine ≥ 3 mg/dl) according to renal histological findings found in kidney biopsy of MM patients. [file 8859340.f1.pdf]

Supplemental Table 1. Prediction of severe renal impairment (serum creatinine ≥ 3 mg/dl) according to renal histological findings found in kidney biopsy of MM patients

	Cast nephropathy	Deposition Disease	AL amyloidosis	Interstitial nephritis
Patients-n.	30	3	7	4
Severe renal impairment-(%)	29 (96.6)	1 (33.3)	4 (57.1)	2 (50)
AKI requiring RRT	11 (36.6)	0	0	1 (25)
Nephrotic syndrome	0	2 (66.6)	5 (71.4)	0
Prediction of severe renal impairment-(%)	P=0.004*	0.189	0.196	0.181
